# Physical Activity Prevents Cartilage Degradation: A Metabolomics Study Pinpoints the Involvement of Vitamin B6

**DOI:** 10.3390/cells8111374

**Published:** 2019-11-01

**Authors:** Michela Deiana, Giovanni Malerba, Luca Dalle Carbonare, Samuele Cheri, Cristina Patuzzo, Grygoriy Tsenov, Lucas Moron Dalla Tor, Antonio Mori, Gianantonio Saviola, Donato Zipeto, Federico Schena, Monica Mottes, Maria Teresa Valenti

**Affiliations:** 1 Department of Medicine, Internal Medicine, Section D, University of Verona, I-37134 Verona, Italy; michela.deiana@univr.it (M.D.); luca.dallecarbonare@univr.it (L.D.C.); samuele.cheri@univr.it (S.C.); 2 Department of Neurosciences, Biomedicine and Movement Sciences, University of Verona, I-37134 Verona, Italy; giovanni.malerba@univr.it (G.M.); cristina.patuzzo@univr.it (C.P.); grygoriy.tsenov@univr.it (G.T.); lucas.morondallator@univr.it (L.M.D.T.); antonio.mori@univr.it (A.M.); donato.zipeto@univr.it (D.Z.); federico.schena@univr.it (F.S.); monica.mottes@univr.it (M.M.); 3 Istituti Clinici Scientifici Maugeri IRCCS, Rheumatology and Rehabilitation Uniti of the Institute of Castel Goffredo, I-46042 Mantua, Italy; gianantonio.saviola@icsmaugeri.it

**Keywords:** physical activity, cartilage, osteoarthritis, metabolomics, SOX9, vit.B6

## Abstract

Osteoarthritis (OA) is predominantly characterized by the progressive degradation of articular cartilage, the connective tissue produced by chondrocytes, due to an imbalance between anabolic and catabolic processes. In addition, physical activity (PA) is recognized as an important tool for counteracting OA. To evaluate PA effects on the chondrocyte lineage, we analyzed the expression of SOX9, COL2A1, and COMP in circulating progenitor cells following a half marathon (HM) performance. Therefore, we studied in-depth the involvement of metabolites affecting chondrocyte lineage, and we compared the metabolomic profile associated with PA by analyzing runners’ sera before and after HM performance. Interestingly, this study highlighted that metabolites involved in vitamin B6 salvage, such as pyridoxal 5′-phosphate and pyridoxamine 5′-phosphate, were highly modulated. To evaluate the effects of vitamin B6 in cartilage cells, we treated differentiated mesenchymal stem cells and the SW1353 chondrosarcoma cell line with vitamin B6 in the presence of IL1β, the inflammatory cytokine involved in OA. Our study describes, for the first time, the modulation of the vitamin B6 salvage pathway following PA and suggests a protective role of PA in OA through modulation of this pathway.

## 1. Introduction

Osteoarthritis (OA) is a chronic, age-related degenerative disease of articular cartilage and is associated with dramatic changes in cartilage homeostasis. Commonly affected body areas are the knee, hip, hand, spine, and foot. Women are more frequently affected by OA, especially after the age of 50. Disease progression is associated with pain and disability, with a considerable socio-economic impact [1]. An imbalance between degradation and synthesis of cartilage, ruled by chondrocytes, occurs in OA [2]. Chondrogenesis depends on mesenchymal stem cell (MSC) condensation and chondroprogenitor cell differentiation following the upregulation of SOX9, the master transcription factor for MSC differentiation into chondrocytes [3]. Chondrocytes maintain cartilage homeostasis by regulating the replacement of specific matrix proteins. However, during aging and joint disorders, the loss-rate of matrix compounds such as collagens and proteoglycans may exceed the deposition-rate of newly synthesized molecules, thus affecting homeostasis [4]. However, when this balance is disrupted, cartilage degradation elicits inflammation, with consequent typical OA pain.

Physical exercise (i.e., strengthening exercise and general aerobic exercise) has been suggested to improve joint function and to reduce pain [5]; guidelines related to the type and frequency of exercise have been reported [6]. In a previous study, we observed that half-marathon (HM) improves bone differentiation by inducing the upregulation of the osteogenic master gene RUNX2 in progenitor cells treated with sera collected from runners after physical performance, and modulates different molecular pathways involved in immune response, lipid transport, and coagulation [7]. Moreover, exercise positively modulates molecular pathways related to cellular and tissues homeostasis; a positive role of physical activity for cartilage and bone health has been reported as well [8,9]. Recently, we demonstrated that physical exercise enhances the expression of the chondrogenic transcription factor SOX9 by inducing the autophagy process in mesenchymal stem cells. Yet, effects of physical activity are poorly understood at molecular levels. In this study, we evaluated how HM may impact on the chondrocyte commitment of circulating progenitor cells. Next, we mapped the metabolomic profile in runners before (PRE) and after (POST) the competition. The analysis identified several modulated metabolites, including pyridoxal 5′-phosphate and pyridoxamine 5′-phosphate, that are involved in vitamin B6 salvage.

Hence, in order to explore the protective role of vitamin B6 in cartilage, we analyzed the effects of vitamin B6 treatment at molecular and cellular levels in two different OA models (differentiated chondrocytes and SW1353 chondrosarcoma cells). Interestingly, vitamin B6 upregulated SOX9, as well as genes encoding cartilage ECM components, and counteracted the negative effects of ILβ1 in an in vitro model mimicking OA.

These results suggest, for the first time, that physical activity protects chondrocyte lineage and cartilage integrity promoting vitamin B6 activity.

## 2. Materials and Methods

### 2.1. Subjects

Six male amateur runners were enrolled during a sport event called ‘Run for Science’, held in Verona (Italy) in April 2016. The runners were recruited as we previously reported [10]. The runners (median age 40.2 ± 8 years) carried out a 21.1 km half marathon. Written informed consent was obtained from all participants and the study was approved by the Ethical Committee of Azienda Ospedaliera Universitaria Integrata of Verona, Italy (approval number 1538).

### 2.2. Circulating Progenitor Cells (CPCs)

CPCs were isolated from 25 mL of heparinized blood using a depletion method of hematopoietic cells, as we previously reported [11]. In particular, CPCs were collected from heparinized blood by two Ficoll procedures to remove hematopoietic cells using an antibodies cocktail. Briefly, in the first Ficoll procedure, a Rosette-antibody cocktail (against CD3, CD14, CD19, CD38, and CD66b positive cells) was incubated with samples for 20 min at room temperature. Then, a second Ficoll procedure was performed to remove hematopoietic cells crosslinked to red blood cells (glycophorin A). Gene expression analysis for CD3, CD14, CD19, CD45, CD34, CD73, and CD105 markers, as reported previously [12], was conducted to identify cell phenotype.

### 2.3. Sera Collection

Peripheral blood samples were collected before and immediately after the run. Sera were obtained from 10 mL of fresh blood by centrifugation at 1800× *g* for 15 min at 4 °C. Then, sera were harvested and frozen in aliquots at −80 °C until use.

### 2.4. Metabolomics

Sample preparation was performed according to standard protocols [13].

MS setup: Serum metabolites were detected using liquid chromatography combined with electrospray ionization tandem mass spectrometry (HPLC–ESI-MS/MS). The analytic system consisted of Accela 1250 pump, Accela autosampler, and LTQ Orbitrap Velos mass spectrometer (Thermo Scientific, USA). Analytes were separated on Kinetex column C18 100 mm × 2.1 mm × 1.7 µm and mobile phase (solvent A: Aqueous solution of acetic acid pH 2; solvent B: Methanol) in gradient elution at a flow rate of 300 µL/min. The column temperature was maintained at 25 °C; the HPLC elution program was as follows: 5% methanol (2 min), 30% methanol (linear increase in 1 min), 30% methanol (5 min), 5% methanol (linear decrease in 1 min), 5% methanol (3 min). Each sample was measured in triplicate and single injection volume was 5 µL. Metabolites were detected both in the positive (ESI+) and in the negative (ESI−) ionization mode as previously reported [14].

Raw data processing: Raw MS data files were processed using XCMS software Version 3.2.7.1. (The Scripps Research Institute, North Torrey Pines Road BCC-007, La Jolla, CA 92037, USA) Features were associated to known metabolites, when possible, searching for their M/Z and RT values in the Metlin database.

Features presenting a missing value rate >20% were removed. Variables showing a low variation and outlier values were removed through filtering based on interquartile range (IQR). Each feature was normalized by median-normalization and scaled by auto scaling (mean-centered and divided by the standard deviation), as previously reported [15,16].

### 2.5. XTT Test

Cell viability was evaluated after the addition of the effectors by the reduction of the tetrazolium salt XTT (sodium 3I-[1-phenylamino-carbonyl-3,4-tetrazolium]-bis(4-methoxy-6-nitro) benzene sulfonic acid hydrate Cell proliferation kit II—XTT Chemicon), as previously reported [17].

Eight replicas in three independent experiments were tested.

### 2.6. Cell Cultures

Mesenchimal stem cells (PromoCell, Heidelberg, Germany) were plated at a density of 5 × 10^4^ cells per well on 24-well plates and cultured with mesenchimal stem cell growth medium (PromoCell). After 24 h, the chondrogenic differentiation medium (mesenchymal stem cell chondrogenic differentiation medium containing sodium pyruvate, TGFβ3, dexamethasone and 2-phospho ascorbate, (PromoCell) was added and then plates were incubated at 37 °C in a humidified atmosphere with 5% CO_2_. Differentiating cells were cultured for 21 days and then used for further analyses. The chondrosarcoma SW1353 cells (PromoCell) were plated at a density of 5 × 10^4^ cells per well and cultured in the presence of DMEM 10% FBS medium at 37 °C with 5% CO_2_.

After 24 h, IL1β, an inflammatory cytokine, was added to both culture media in order to mimic OA conditions, as previously reported [18]. Pyridoxal hydrochloride (vitamin B6, Sigma, Darmstadt, Germany) was prepared following manufacturer’s instructions and as previously reported [18]. To identify the final concentration of the effectors used in cultures, we performed an XTT analysis, testing different concentrations (for vitamin B6, 300, 200, 100, 50, and 25 µM, while for IL1β, 5, 1, and 0.5 ng/mL). The IC50 determined to evaluate the toxicity for vitamin B6 as 251.4 µM for SW1353 cells, while the concentrations used for assaying the IC50 in MSCs did not affect the viability for MSCs (IC50 > 300 µM). The IC50 for IL1β were 6.3 ng/µL and 4.6 ng/µL for SW1353 and MSCs, respectively. Therefore, we chose to use the concentrations of 100 μM and 1 ng/mL (concentrations below of the toxicity levels evaluated by IC50) of vitamin B6 and IL1β, respectively, according to our results and as previously reported [19,20]. Three different combinations of supplements were added to the cell cultures during chondrogenic differentiation or proliferation. In detail: IL1β (ng/mL) alone, Vitamin B6 (100 μM) alone, IL1β + Vitamin B6. Cultures were incubated for 8 days. Cultures without supplements were used as controls. Three independent experiments were performed for each condition.

### 2.7. TUNEL Assay

The TUNEL technique (ApoTag Fluorescein In Situ Apoptosis Detection Kit, S7110, Millipore Corporation, Billerica, MA, USA) was used to analyze DNA damages due to apoptosis in a cell culture plated on culture slide, as previously reported [21]. Four different fields were measured for each sample, in three independent experiments with about 80–100 total cells.

### 2.8. Senescence Assay

Senescence levels were measured using the Senescence detection kit (Abcam, Cambridge, UK) according to manufacturer’s instructions. Cells were observed under a DMi1 microscope (Leica, Wetzlar, Germany) at 200× total magnification.

### 2.9. Cellular Reactive Oxygen Species (ROS) Detection

ROS were measured by staining cells with the DCFDA cellular ROS detection assay kit (Abcam) according to the manufacturer’s protocol. After the staining procedures, cells were analyzed measuring fluorescence (ex/em = 485/535 nm) in end point at the VictorX4 instrument (Perkin Elmer, Milan, Italy).

### 2.10. Total RNA Extraction

The RNA assay Minikit (Quiagen, Hilden, Germany) with DNAse I treatment was used to extract total RNA, as previously reported [22]. RNA obtained from differentiated MSCs, SW1353, and CPCs was then quantified spectrophotometrically. The RNA preparation was considered pure when the 260/280 nm absorbance ratio was in the 1.8 to 2.0 range. For gene expression studies related to CPCs, RNA samples were pooled across the six subjects in order to reduce the bias that each individual might introduce.

### 2.11. Reverse Transcription

First-strand cDNA complementary to mRNA was generated using the First Strand cDNA Synthesis Kit (GE Healthcare, Chicago, IL, USA) according to manufacturer’s protocol. RT products were aliquoted in equal volumes and then stored at −80 °C.

### 2.12. Real Time RT-PCR

PCR was performed in a total volume of 20 µL containing 1X Taqman Universal PCR Master Mix, no AmpErase UNG, and 2 µL of cDNA from each sample; the following pre-designed, specific primers and probe set was obtained from Assay-on-Demand Gene Expression (Thermofisher Scientific, Waltham, MA, USA): COX2 Hs00153133_m1, SOX9 Hs00165814_m1, COMP Hs00164359_m1, COL2A1 Hs00264051_m1, and β2M Hs00187842_m1. The real-time amplifications included 10 min at 95 °C, followed by 40 cycles at 95 °C for 15 sec and at 60 °C for 1 min. Thermocycling and signal detection were performed with the ABI Prism 7300 Sequence Detector and signals were detected, as previously reported [23]. The expression levels were calculated for each sample in triplicate after normalization against the housekeeping gene (β_2_-microglobulin), using relative fold expression differences.

### 2.13. Alcian Blue Staining

Alcian blue staining was performed as previously described [24]. Briefly, cell slides were fixed with 95% PFA and then stained with 1% Alcian blue 8GX HCl overnight. The following day, cell slides were washed and observed under microscope.

### 2.14. Statistical and Bioinformatics Analyses

Non-parametric tests were used when the statistical test was applied to small size samples, since, in such cases (for instance in PRE and POST runs), normal distribution of the data cannot be ascertained with confidence. Differences among groups were tested using the non-parametric Wilcoxon test (2 groups) or Kruskal–Wallis test (more than 2 groups), together with the Dunn’s multiple comparison test as “post-hoc” procedure. Metabolomic dysregulated features (fold change ≥1.5 and nominal *p*-value ≤0.05, arbitrarily chosen) were plotted in a cloud plot, reporting intensities of signals between groups. Statistical analysis of metabolite-associated features was performed using XCMS and MetaboAnalyst (v.4.0) [16]. Statistical analyses for in vitro experiments were performed using the R software (v. 3.5, R core team, Vienna, Austria). The enrichment analysis of the biological process was performed using information taken from the Kegg database. Associations were considered statistically significant when the nominal *p*-value was ≤0.05.

## 3. Results

### 3.1. Characterization of CPC Phenotype

RNA was extracted from CPCs isolated from 6 athletes PRE and POST HM. RNA samples were used to evaluate the effective selection of circulating progenitors by analyzing the expression of the specific markers [25]. As the amount of circulating progenitors is very low [26], we pooled the single runners’ CPC RNA to obtain sufficient material for the analyses and to avoid potential individual bias.

Table 1 shows the expression of cluster differentiation (CD) of mesenchymal cell phenotype (CD 105 and CD 73), as well as of hematopoietic cells, in pooled CPC mRNA obtained before (PRE) and after (POST) the run.

### 3.2. Overexpression of Chondrogenic Genes in POST Run CPCs

**Figure 1:**
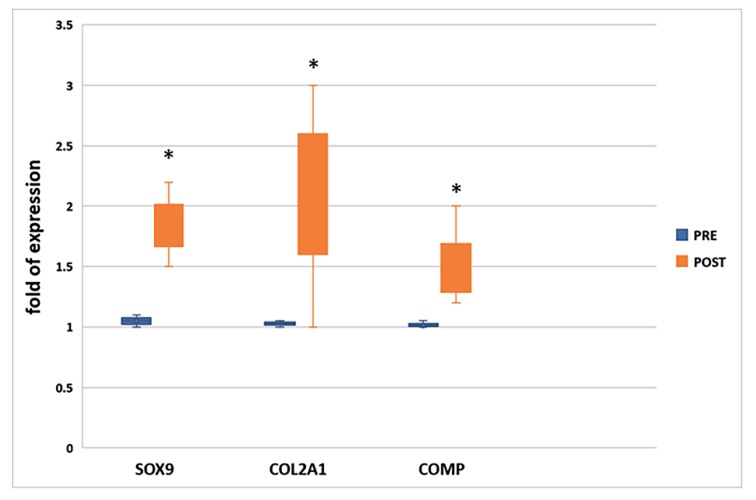
Chondrogenic transcription factor SOX9, as well as COL2A1 and COMP, were upregulated (fold of gene expression >1) in CPCs collected after the run (POST) * *p* ≤ 0.05 vs. PRE.

To evaluate the effects of physical activity on the chondrogenic commitment and differentiation, we analyzed the expression of the chondrogenic transcription factor SOX9 and of genes involved in cartilage production (COL2A1 and COMP) in pooled CPC mRNA obtained before (PRE) and after (POST) the run. As shown in Figure 1, all genes involved in the chondrogenic lineage were upregulated after the run.

### 3.3. Metabolomic Profile

**Figure 2:**
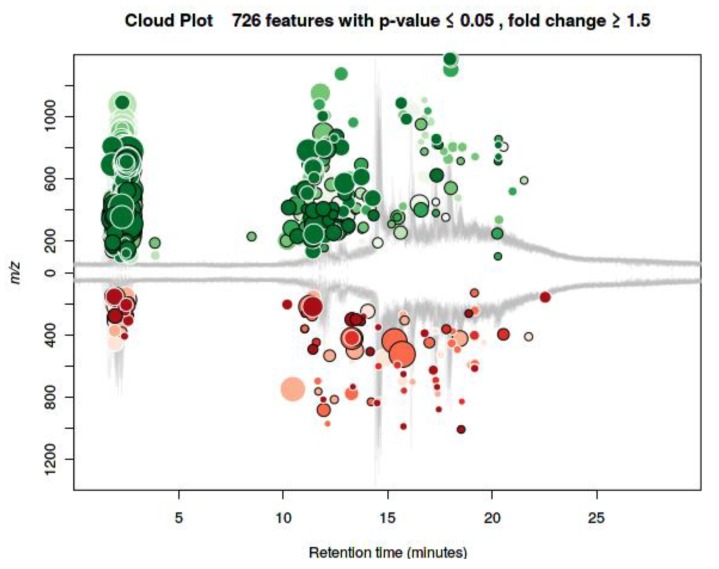
Cloud plot of the metabolomics modulated features (fold change ≥ 1.5, *p*-value ≤ 0.05). Feature signals belonging to molecules defined by m/z and retention time. Differential analysis based on the intensity of the signals in PRE versus POST run sera. Green and red circles represent the upregulated and downregulated features, respectively. The size value of each circle corresponds to (log) fold change. Color shades are used to represent the *p*-value, with brighter circles indicating lower *p*-values. The retention time corrected (TIC) total ion chromatograms are overlaid in gray in the figure background.

To evaluate the association of metabolites with chondrogenic differentiation, we performed metabolomic analysis of individual serum collected before and after the run. Untargeted metabolomics profiling (HPLC-Orbitrap platform) identified ~5000 features, and 726 of them appeared to be modulated (absolute log fold-change ≥1.5 and nominal *p*-value ≤0.05), as shown in the cloud plot in Figure 2. The metabolomic profile shows, after the run, a general upregulation of several metabolites, given the higher number of green circles compared to the red ones.

**Figure 3:**
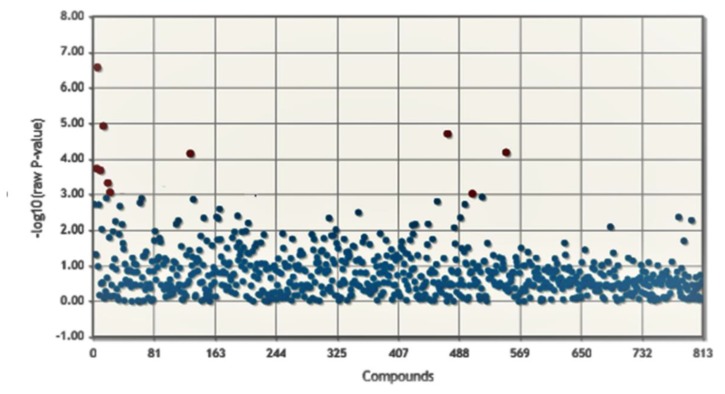
Modulated metabolites between PRE and POST run athletes. The *t*-test was considered as significant only for features with an adjusted *p*-value ≤0.05 (red circles).

To determine if significant differences between the two groups were present, we performed *t*-test analysis. Figure 3 shows the association values for each metabolite in pre- and post-run conditions (log10(adjusted-*p*-value) ≤ 0.05). This analysis identified ten significant modulated metabolites between PRE and POST conditions (red dots in the plot); details are reported in Table 2. In Supplementary Materials Table S1, the results of the first 100 features are reported.

Significantly modulated metabolites were used to perform enrichment analysis of the biological process according to the Kegg database.

**Figure 4:**
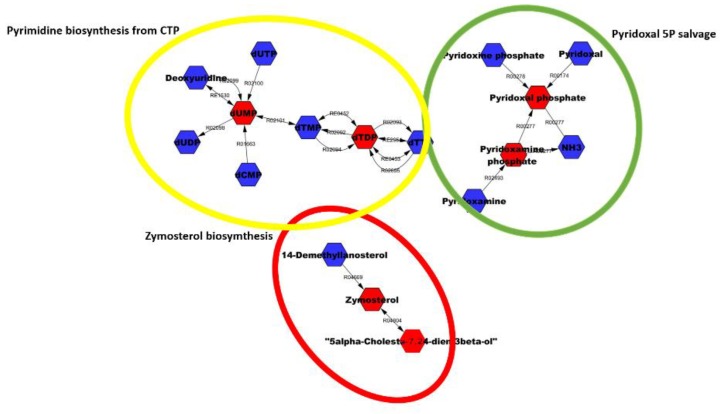
The most significantly modulated terms suggested by metabolomic analysis between the two groups.

Table 3 shows six highly modulated metabolites identified on the basis of the putative overlapping ones. We noted that among the 10 significant metabolites reported in Table 2, only six were present in the list of metabolites, belonging to at least one of the three enriched KEGG terms. In detail, two belong to pyrimidine deoxyribonucleotides biosynthesis from CTP (dUMP, dTDP); two to the pyridoxal 5 phosphate salvage (pyridoxal 5′-phosphate, pyridoxamine 5′-phosphate), and two to the zymosterol biosynthesis (4α-formyl-4β-methyl-5α-cholesta-8,24-dien-3β-ol, 4,4-dimethylzymosterol). Interestingly, Pyridoxal 5′-phosphate (PLP) is the active form of vitamin B6, whereas pyridoxine and its phosphate ester and pyridoxamine 5′-phosphate form the vitamin B6 complex. Figure 4 shows the significantly modulated terms (details in Table 3); metabolites in red represents the significantly modulated metabolites identified in the athletes after HM; blue metabolites depict the other metabolites forming that pathway.

### 3.4. Vitamin B6 Upregulates the Expression of Genes Related to Chondrogenic Differentiation and Stimulates Chondrocyte Maturation

**Figure 5:**
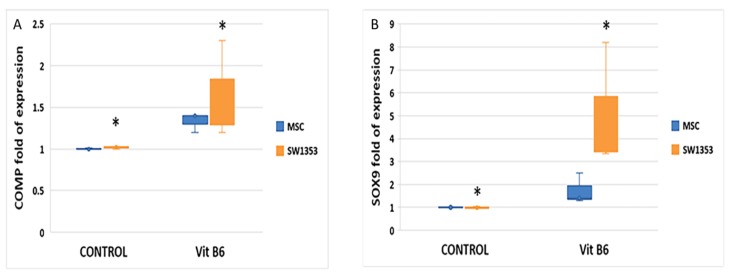
Vitamin B6 (100 µM) increased the expression levels of the chondrogenic transcription factor SOX9 (**A**) and of COMP (**B**) in both MSC and SW1353 cell lines; * *p* ≤ 0.05 vs. controls.

Among the metabolites identified, we investigated the role of vitamin B6 in chondrogenesis. Therefore, we analyzed the effects of vitamin B6 supplementation in mesenchymal stem cells (MSCs) during chondrogenic differentiation and in human chondrosarcoma SW-1353 cells, mimicking the properties of immature chondrogenic cells [27]. To evaluate toxicity and assess the optimal concentration of vitamin B6, we performed a XTT study; results are reported in the Supplementary Material (Supplementary Figures S1A and S2A). As shown in Figure 5, gene expression levels of the chondrogenic transcription factor SOX9 (A), as well as of the ECM component COMP (B), were increased in both vitamin B6-treated cell lines compared to controls (untreated samples).

### 3.5. Vitamin B6 Counteracts the Negative Effects of IL1β in MSCs during Chondrogenic Differentiation

**Figure 6:**
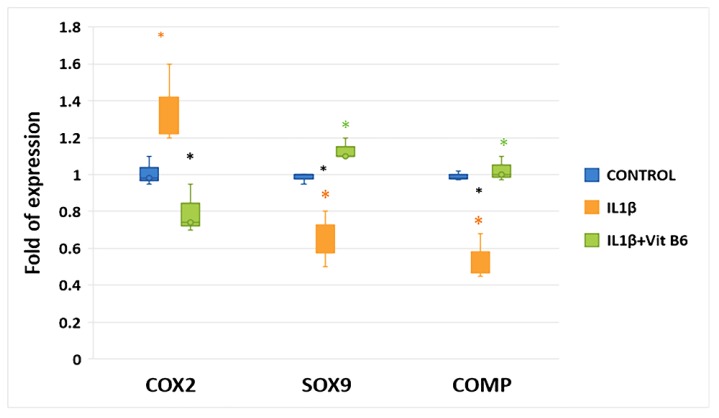
The presence of 1 ng/mL IL1β caused an increase of the inflammatory factor COX2 gene expression and reduced SOX9 and COMP genes expression during chondrogenic differentiation. Vitamin B6 supplement (100 µM) was able to revert IL1β effects on MSCs during chondrogenic differentiation. * *p* ≤ 0.05, (* CTRL vs. IL1β, * CTRL vs. IL1β+ Vit B6, * IL1β vs. IL1β+ Vit B6).

As performed for vitamin B6, we conducted a dose-response test in cells treated with IL1β; results are shown in the Supplementary Materials (Supplementary Figure S2A,B). MSCs were cultured in chondrogenic differentiating medium with or without 1 ng/mL of IL1β, an inflammatory cytokine involved in OA pathogenesis. IL1β-treated MSCs showed higher levels of the inflammatory factor cyclooxygenase COX2 gene expression compared to controls, while the expression of both SOX9 and COMP genes was reduced (Figure 6). The addition of 100 µM vitamin B6 to cultures was able to reduce COX2 gene expression in IL1β-treated MSCs. Furthermore, the addition of vitamin B6 restored SOX9 and COMP genes expression in IL1β-treated MSCs (Figure 6).

### 3.6. Vitamin B6 Counteracts the Negative Effects in an In Vitro OA Model

**Figure 7:**
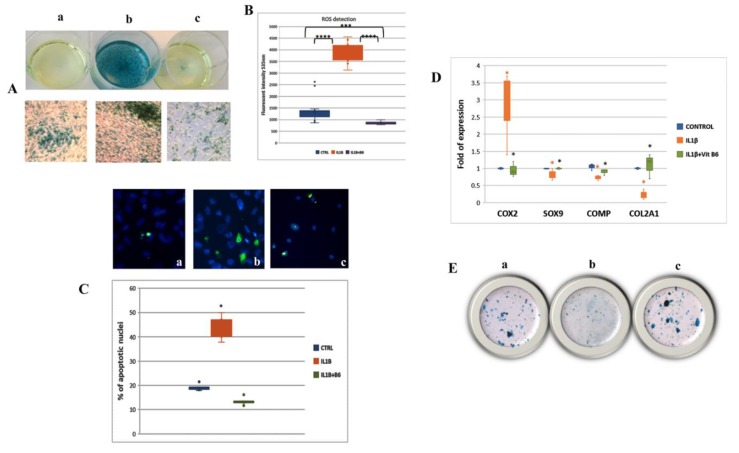
(**A**) Senescence-associated beta-galactosidase staining was increased in SW1353 cells treated with IL1β (**b**) compared to controls (**a**); vitamin B6′s supplementation counteracted IL1β-induced senescence (**c**). (**B**) The generation of ROS induced by IL1β in SW1353 was reduced by adding vitamin B6 in cells medium. (**C**) Similarly, vitamin B6′s supplementation (**c**) was able to reduce IL1β-induced apoptosis (**b**) in SW1353 cells. (**D**) The increased COX2 gene expression and the reduced expression of chondrogenic genes due to IL1β treatment were reverted thanks to vitamin B6 supplementation. (**E**) Vitamin B6 ability in counteracting IL1β effects in SW1353 was confirmed by Alcian blue staining evaluating GAG production: (**a**) Controls; (**b**) IL1β treated; (**c**) IL1β + vit B6 treated; magnifications: A: 5X (Well plate) and 10X (culture slides); C: 20X; E: 5X; * *p* ≤ 0.05; *** *p* ≤ 0.0005; **** *p* ≤ 0.000005. (* CTRL vs. IL1β, * IL1β vs. IL1β+ Vit B6).

As previously reported, SW-1353 cells treated with IL1β mimic the phenotype of primary chondrocytes from OA patients [28]. In fact, the senescence-associated beta-galactosidase staining, as well as the generation of ROS and the number of apoptotic cells, increased in SW-1353 cell cultures supplemented with IL1β (Figure 7A,B). However, vitamin B6 addition to IL1β-treated cells was able to revert the cellular phenotype. The expression of the inflammatory factor COX2 gene dropped, while the expression of genes involved in chondrocyte differentiation and maturation, such as SOX9, COMP, and COL2A1, increased in IL1β-treated cells in the presence of vitamin B6 (Figure 7C).

In addition, Alcian blue staining, showing the production of glycosaminoglycan (GAG), further demonstrating the ability of vitamin B6 to counteract the negative effects of IL1β (Figure 7D).

## 4. Discussion

Physical activity can prevent cartilage disorders and it has been suggested as a therapeutic tool for individuals with osteoarthritis (OA) [29]. Andersson et al. reported that COMP serum levels increase after physical activity in OA patients [30]. However, the molecular pathways modulated by physical activity have been poorly investigated so far. Notably, in recent times, metabolomics has arisen as a powerful technique to study body metabolism. In fact, metabolites represent actual products of biochemical and cellular activity [31]. In our study, we evaluated the effects of physical activity (e.g., HM) on the chondrocyte lineage at several levels, including gene expression, metabolomics, cell senescence, apoptosis, and oxidative stress.

Interestingly, the expression of genes involved in commitment (SOX9) and maturation (COL2A1, COMP) of the chondrocyte lineage was upregulated in POST run circulating progenitor cells, and these data confirmed our previous study conducted in 22 individuals [10].

In addition, metabolomics showed that different pathways, such as pyrimidine (from CTP) zymosterol biosynthesis, and vitamin B6 salvage, were modulated in runners’ sera.

Pyrimidine nucleotide availability is important for regulating the proliferation process in mammalian cells. Interestingly, it has been demonstrated that rats treated with cytosine monophosphate (CMP) and uridine monophosphate (UMP) can endure longer periods of exercise [32]. Therefore, the increased pyrimidine synthesis in POST run sera can be explained as a way to enhance physical endurance.

In POST run sera, we observed a significant modulation of metabolites involved in the biosynthesis of zymosterol, a precursor of cholesterol. In particular, we observed the upregulation of 4,4-dimethylzymosterol, while the 4α-formyl-4β-methyl-5α-cholesta-8,24-dien-3β-ol was downregulated in POST run sera. This modulation can be explained by considering that many intermediates, in particular the sterols, regulate cholesterol synthesis [33]. Previous studies reported the effects of physical activity on cholesterol and lipid profile; increased HDL/LDL cholesterol ratio following exercise has been reported [34]. However, as hypoxia affects lipid metabolism [35], the modulation of zymosterol biosynthesis might be a consequence of hypoxia induced by the oxidative stress associated with physical activity [36]. Similarly, the downregulation of lipoic acid, an antioxidant compound [37] that we observed in POST run sera, may be due to its consumption aimed at counteracting PA-induced oxidative stress.

Interestingly, we observed two metabolites involved in vitamin B6 salvage (pyridoxal 5′-phosphate, pyridoxamine 5′-phosphate), showing increased levels in POST competition runners. Vitamin B6 is a co-factor that plays a crucial role in several metabolic functions in humans [38]. The biosynthesis of vitamin B6 mainly occurs in plants and microorganisms. Mammals cannot synthesize vitamin B6, but they can obtain it from two different sources: Dietary intake and bacteria in the gut [39]. It has been demonstrated that gut bacteria produce vitamin B6, making it available for the host [40]. Importantly, it has been shown that PA can promote wellness, influencing or interacting with the gut microbiota [41]. Recent studies suggest that PA can raise the number of beneficial microbial strains, promote the microflora diversity, and enhance the development of commensal bacteria [42], inducing changes in the bacterial flora composition, enhancing the biosynthesis of molecules that can exert anti-inflammatory functions, activation of the hypothalamic–pituitary–adrenal (HPA) axis, and reinforcement of neuromuscular function [43]. Said et al. have recently elucidated the mechanism of vitamin B6 uptake in the large intestine [39]. On the basis of our results, we hypothesize that physical activity may prevent OA, as well as other chronic diseases, by inducing gut microbes to produce vitamin B6. Certainly, the concurrent association between PA, vitamin B6, and microbiome is intriguing and highly speculative. Further studies with larger sample sizes are needed to explore this hypothesis.

Furthermore, vitamin B6 has been reported to reduce negative effects caused by ROS through the inhibition of the xanthine oxidase activity [44]. Therefore, vitamin B6 may protect cartilage by controlling the oxidative stress which is known to play an important role in several diseases, including OA [45].

The increased pyridoxal 5′-phosphate and pyridoxamine 5′-phosphate in POST run sera suggests that physical activity could prevent cartilage disorders by increasing the uptake of vitamin B6.

Pyridoxal 5′-phosphate (PLP), the active form of vitamin B6, is involved in more than 150 enzymatic reactions as a co-factor; low levels of PLP are associated with different chronic diseases [46]. In particular, low vitamin B6 levels have been reported in cardiovascular diseases [47], cancer [46], inflammatory bowel disease [48], and diabetes [49]. Previous studies demonstrated that reduced levels of pyridoxal 5′-phosphate (PLP) are associated with pro-inflammatory levels of cytokines [50,51]. It has been reported that vitamin B6 supplementation (100 mg/day) reduces pro-inflammatory cytokine levels in patients affected by rheumatoid arthritis [52]. Conversely, vitamin B6 deficiency induced cartilage disorders resembling osteoarthritis features in experimental models [53]. Recently, increased levels of miRNAs targeting vitamin B6 metabolic pathways have been reported in Kashin–Beck disease (KBD), a chronic osteochondropathy [54]. 

The in vitro results reported in the present study suggest a role for vitamin B6 in promoting chondrocyte differentiation and maturation. Previous studies tested different concentrations of vitamin B6, ranging from 5 to 500 µM, in in vitro experiments [55,56]. In our experiments, we treated cells with a 100 µM vitamin B6 concentration. This concentration was not toxic (as resulting by calculating the IC50), even if it was able to modulate cell viability in SW1353. Therefore, we observed a higher gene expression of SOX9, COL2A1, and COMP, the genes associated with chondrocyte maturation, in MSCs and chondrosarcoma cells treated with vitamin B6. In addition, vitamin B6 was observed to counteract IL1β negative effects. Vitamin B6, together with IL1β, restored the expression levels of SOX9 and COMP and reduced the expression levels of the inflammatory factor COX2. This was also observed in immature chondrocytes. The more abundant GAG production and the reduced apoptosis and ROS levels in IL1β-treated chondrocytes confirm the anti-inflammatory effect of vitamin B6 and its protective role for chondrocytes in an OA-mimicking model.

## 5. Conclusions

In this study, we observed the modulation of the metabolomic profile related to vitamin B6 salvage and the promotion of chondrocyte differentiation following physical activity. We also demonstrated that vitamin B6 can counteract chondrocyte injuries in an OA model.

In conclusion, the present study highlights, for the first time, vitamin B6 salvage modulation upon physical activity for the prevention of OA-related diseases.

## Figures and Tables

**Table 1:** Relative expression (in percent) of cluster differentiation (CD) obtained by depletion method in PRE and POST run CPCs.

Cluster Differentiation Transcript	Pre Run (%) (Media and SD)	Post Run (%) (Media and SD)	*p*-Value
CD105	68 ± 0.3	67 ± 0.4	0.2
CD73	73 ± 0.2	72 ± 0.3	0.06
CD3	0	0	NA *
CD14	0.5 ± 0.07	0.6 ± 0.08	0.12
CD19	0	0	NA *
CD45	1.6 ± 0.3	1.8 ± 0.3	0.06
CD34	Low levels	Low levels	NA *

* NA, not available.

**Table 2:** List of significantly modulated metabolites.

Name	*p*-Adjusted	Nominal *p*-Value	Log2fold	Modulation (Up/Down)
Pyridoxamine 5′-phosphate	0.0003	0.0000072958	5.4	UP
Lipoic acid. reduced	0.0003	0.0000076800	−1.56	DOWN
Pyridoxal 5′-phosphate	0.0006	0.0000000104	4.1	UP
dUMP	0.0007	0.0000192660	5.93	UP
4.4-dimethylzymosterol	0.004	0.0000397850	2.72	UP
1.3-Dimethyl-8-phenylxanthine	0.012	0.0000458276	7.16	UP
Phosphatidylinositol-3.4.5-trisphosphate	0.017	0.0000662191	1.56	UP
4α-formyl-4β-methyl-5α-cholesta-8.24-dien-3β-ol dTDP	0.019 0.021	0.0000815126 0.0000837270	−4.94 60.4	DOWN UP
8-oxo-dGTP	0.049	0.0000930718	−1.79	DOWN

**Table 3:** Pathways identified by highly modulated metabolites between PRE run and POST run sera.

Pathway	All Metabolites	Overlapping Metabolites	*p*-Value (Raw)	*p*-Adjusted
Pyrimidine deoxyribonucleotides biosynthesis from CTP	2	2	0.0094	0.0361
Pyridoxal 5-phosphate salvage	4	2	0.031	0.1164
Zymosterol biosynthesis	5	2	0.05	0.244

## Supplementary Materials

The following are available online at https://www.mdpi.com/2073-4409/8/11/1374/s1, Figure S1: XTT analysis of vitamin B6 (A) and IL1β (B) concentration toxicity in MSC model. P-values calculated using a Wilcox non parametric test are reported above each concentration obtained from the comparison versus the control. Figure S2: XTT analysis of vitamin B6 (A) and IL1β (B) concentration toxicity in chondrosarcoma model. P-values calculated using a Wilcox non parametric test are reported above each concentration obtained from the comparison versus the control. Table S1: t-test analysis of t-test analysis of modulated metabolites PRE and POST HM. In teable are reported only the 100 modulated features, among 726 identified and reported in the cloud plot.

## Abbreviation

CD	Cluster of differentiation
CMP	Cytosine monophosphate
CO_2_	Carbon dioxide
COL2A1	Collagen Type II Alpha 1 Chain
COMP	Cartilage Oligomeric Matrix Protein
COX	Cyclooxygenase 2
CPCs	Circulating progenitor cells
CTP	Cytidine-triphosphate
DMEM	Dulbecco’s Modified Eagle Medium
ECM	Extra cellular matrix
FBS	Fetal bovin serum
GAG	Glycosaminoglycan
HDL	High Density Lipoprotein
HM	Half marathon
IL1β	Interleukin 1 Beta
IQR	Interquartile range
KBP	Kashin–Beck disease
LDL	Low Density Lipoprotein
MSC	Mesenchymal stem cell
OA	Osteoarthritis
PA	Physical activity
PFA	Paraformaldehyde
PLP	Pyridoxal 5′-phosphate
ROS	Reactive oxygen species
RT	Reverse transcription
RUNX2	Runt-Related Transcription Factor 2
SOX9	Transcription Factor SOX-9
TIC	Total ion chromatogram
UMP	Uridine monophosphate
Vit.B6	Vitamin B6
