# Supplementary material for: Physical Activity Prevents Cartilage Degradation: A Metabolomics Study Pinpoints the Involvement of Vitamin B6
Source: Cells. 2019 Nov 1;8(11):1374. doi: 10.3390/cells8111374 (PMC6912200; doi:10.3390/cells8111374)
Supplement: Supplementary file 1 [file cells-08-01374-s001.pdf]

Supplementary Figure 1

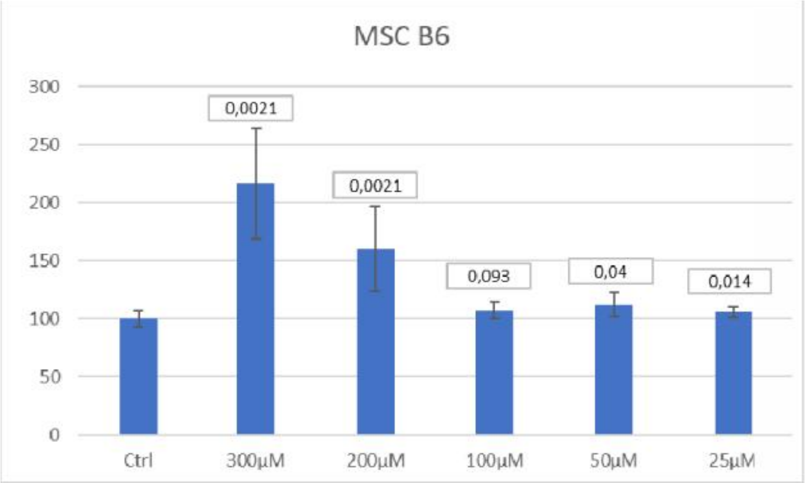

Figure 1A: XTT analysis of vitamin B6 concentration toxicity in MSC model. . P-values calculated using a Wilcox non parametric test are reported above each concentration obtained from the comparison versus the control.

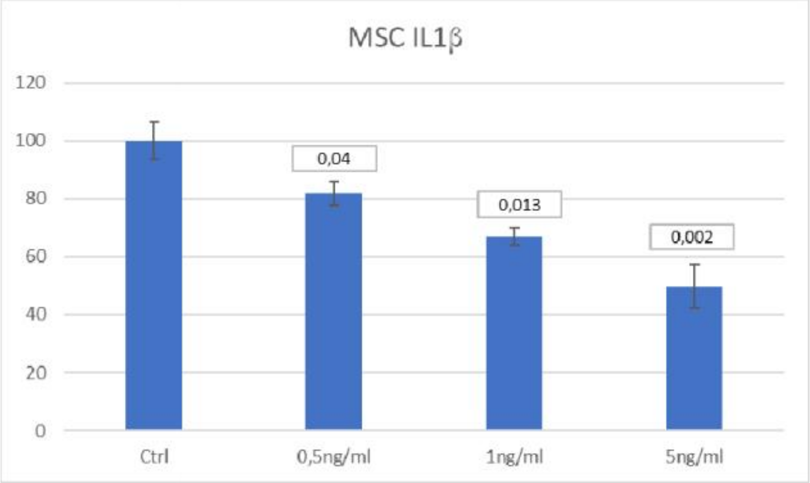

Figure 1B: XTT analysis of IL1β concentration toxicity in MSC model. P-values calculated using a Wilcox non parametric test are reported above each concentration obtained from the comparison versus the control.

Supplementary Figure 2

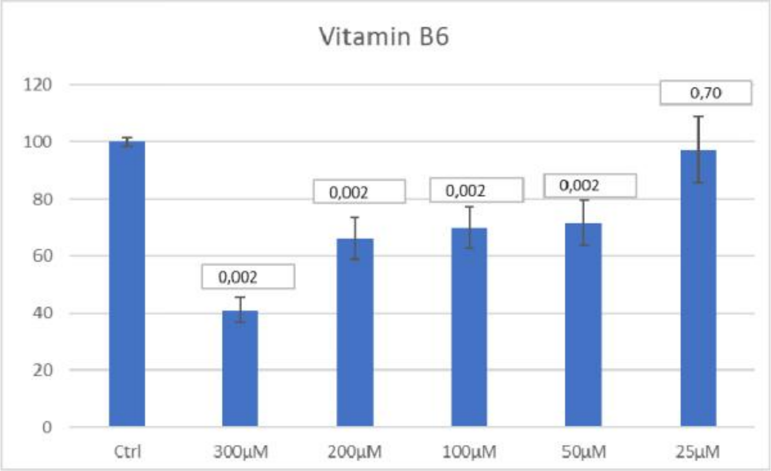

Figure 2A: XTT analysis of vitamin B6 concentration toxicity in chondrosarcoma model. P-values calculated using a Wilcox non parametric test are reported above each concentration obtained from the comparison versus the control.

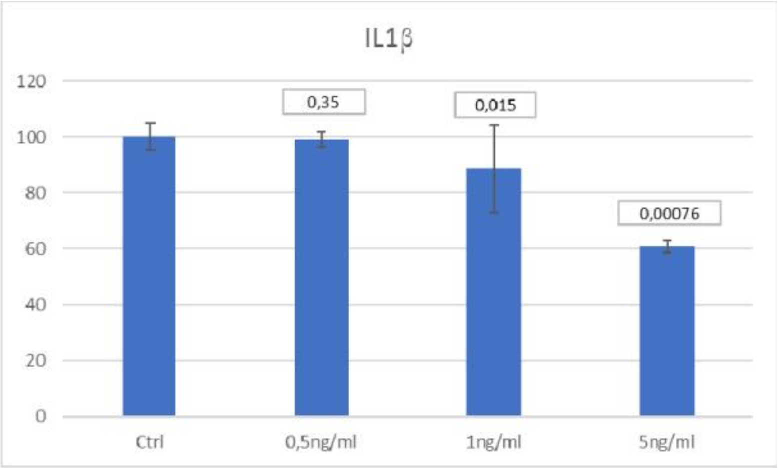

Figure 2B: XTT analysis of IL1β concentration toxicity in chondrosarcoma model. P-values calculated using a Wilcox non parametric test are reported above each concentration obtained from the comparison versus the control.

Supplementary Table 1: t-test analysis of t-test analysis of modulated metabolites PRE and POST HM. In teable are reported only the 100 modulated features, among 726 identified and reported in the cloud plot.

Name	log2fold	pvalue	qvalue	updown
M170T2_3	4,1	0,0000000104	0,0000545148	UP
M231T3	5,4	0,0000072958	0,0131809142	UP
M189T_4	-1,56309	0,0000076800	0,0134116400	DOWN
M191T2_6	5,93	0,0000192660	0,0247122863	UP
M365T2_7	2,72	0,0000397850	0,0347111485	UP
M611T14_1	7,16	0,0000458276	0,0365416072	UP
M423T2_2	1,56	0,0000662191	0,0409162895	UP
M523T3_2	-4,94	0,0000815126	0,0430915493	DOWN
M423T2_4	60,4	0,0000837270	0,0433554930	UP
M709T3_1	-1,79	0,0000930718	0,0443597501	DOWN
M834T17	0,393053	0,0002351295	0,0517402574	UP
M697T2_1	2,259001	0,0002415243	0,0521119148	UP
M427T14_1	-0,32846	0,0002512425	0,0526497899	DOWN
M344T13	-0,37332	0,0002767439	0,0551468178	DOWN
M469T14_1	-0,40886	0,0002907601	0,0564175936	DOWN
M415T10	2,483044	0,0003137989	0,0587133609	UP
M123T2_2	1,137847	0,0003388809	0,0612198904	UP
M708T2_2	2,296505	0,0004203727	0,0683066459	UP
M605T11	1,739517	0,0004248069	0,0686516002	UP
M475T14_2	3,356004	0,0004681274	0,0718341763	UP
M150T3	2,878599	0,0005016288	0,0740833775	UP
M207T3_2	-1,9964	0,0005341234	0,0761092129	DOWN
M407T12_1	2,497957	0,0005613984	0,0777028373	UP
M698T2	2,224737	0,0005893604	0,0792444685	UP
M835T17	0,332035	0,0006009513	0,0798580097	UP
M370T12	1,00735	0,0006066282	0,0801533030	UP
M232T11_1	2,483794	0,0006236753	0,0810201526	UP
M230T2_1	2,530679	0,0006260577	0,0811389808	UP
M396T11_1	2,775774	0,0006405642	0,0818506784	UP
M672T17	0,311373	0,0008000502	0,0907006402	UP
M1060T17	0,407086	0,0008049561	0,0909413331	UP
M424T2_2	1,959054	0,0008294422	0,0921182185	UP
M191T2_4	1,122368	0,0008442649	0,0928114452	UP
M155T2_4	-3,50512	0,0009228133	0,0962618213	DOWN

M203T3	1,872114	0,0009563817	0,0976311066	UP
M506T11_2	2,434726	0,0009638356	0,0979272527	UP
M967T12_2	-0,49916	0,0010067352	0,0995787243	DOWN
M796T12	3,512163	0,0010166677	0,0999487003	UP
M716T3_1	7,521069	0,0010671636	0,1017620240	UP
M193T2_5	1,832402	0,0010983885	0,1028298097	UP
M381T3_1	2,617175	0,0011088051	0,1031774147	UP
M795T17	-0,22502	0,0011439963	0,1043212882	DOWN
M947T17	0,469948	0,0011724774	0,1052140571	UP
M393T3_1	1,760585	0,0011982197	0,1059968361	UP
M674T2_1	3,837176	0,0012079740	0,1062876710	UP
M295T16_2	0,204862	0,0012460797	0,1073945952	UP
M204T2_2	2,444993	0,0012866918	0,1085254705	UP
M719T2_1	1,92518	0,0012929615	0,1086957643	UP
M674T3	5,621679	0,0013248529	0,1095449257	UP
M281T15_3	0,271291	0,0013389088	0,1099103849	UP
M241T2_1	2,192567	0,0014527006	0,1126859950	UP
M295T2_3	1,78968	0,0014796026	0,1132980944	UP
M255T12_3	2,909412	0,0015098946	0,1139687670	UP
M447T14_1	-0,30821	0,0015181785	0,1141488559	DOWN
M205T10	-1,44697	0,0015468719	0,1147619700	DOWN
M419T13_1	-0,31748	0,0015678618	0,1152002630	DOWN
M398T18	-0,44447	0,0015916508	0,1156869222	DOWN
M185T13_2	0,340109	0,0016547523	0,1169283711	UP
M860T12_2	0,938309	0,0016831391	0,1174646080	UP
M523T2_1	2,286102	0,0016984184	0,1177477885	UP
M526T11	3,610576	0,0017357487	0,1184241661	UP
M363T2_2	1,433716	0,0017676376	0,1189851611	UP
M591T2_4	2,620412	0,0018010928	0,1195577863	UP
M231T12_1	2,378323	0,0018580896	0,1204976610	UP
M904T12	-0,42065	0,0018581764	0,1204990597	DOWN
M189T2_2	2,205857	0,0018705154	0,1206968381	UP
M517T18	-0,41017	0,0019914079	0,1225354985	DOWN
M256T2_2	1,919668	0,0020627193	0,1235422028	UP
M312T3	10,28737	0,0020708222	0,1236532076	UP
M791T12_3	4,30506	0,0020710273	0,1236560094	UP
M307T2_5	1,727217	0,0020964236	0,1239995716	UP
M365T14_2	1,372887	0,0020964307	0,1239996670	UP
M300T13_1	2,091415	0,0021176180	0,1242814021	UP
M351T15_1	-0,67633	0,0021478644	0,1246761090	DOWN

M714T20	0,443243	0,0021747280	0,1250194922	UP
M905T12_3	-0,42023	0,0022676893	0,1261583601	DOWN
M275T2_9	1,255795	0,0022942716	0,1264706347	UP
M697T2_3	2,304006	0,0022948854	0,1264777769	UP
M247T2_2	2,433325	0,0022986440	0,1265214529	UP
M630T2_3	3,111297	0,0023559425	0,1271735819	UP
M237T2_1	1,946404	0,0023644453	0,1272682174	UP
M651T16	-0,6788	0,0023732785	0,1273659593	DOWN
M831T14	-0,33946	0,0023858567	0,1275041468	DOWN
M741T20	0,703275	0,0024176632	0,1278484655	UP
M816T20	0,771119	0,0024338206	0,1280206245	UP
M135T3_1	1,682389	0,0024381909	0,1280668776	UP
M233T3	2,922771	0,0024436836	0,1281248207	UP
